# Senomorphic activity of a combination of niacinamide and hyaluronic acid: correlation with clinical improvement of skin aging

**DOI:** 10.1038/s41598-024-66624-7

**Published:** 2024-07-15

**Authors:** Patrick Bogdanowicz, Paul Bensadoun, Maïté Noizet, Benoît Béganton, Armony Philippe, Sandrine Alvarez-Georges, Gautier Doat, Amélie Tourette, Sandrine Bessou-Touya, Jean-Marc Lemaitre, Hélène Duplan

**Affiliations:** 1 R&D Pierre Fabre Dermo-Cosmétique & Personal Care, Toulouse, France; 2 INSERM IRMB UMR1183, Hôpital Saint Eloi, Université de Montpellier, Montpellier, France; 3 Laboratoires Dermatologiques Avène, Lavaur, France

**Keywords:** Senescence, Transcriptomics

## Abstract

Intrinsic and extrinsic factors, including lifestyle and sun exposure, can contribute to cell senescence, which impairs skin homeostasis, that may in turn lead to skin aging. Senescent cells have a specific secretome, called the senescence-associated secretory phenotype (SASP) that includes MMPs, CXCLs and S100A8/9. Reducing the SASP with senotherapeutics is a promising strategy to reduce skin aging. Here we evaluated the effect of a formula containing niacinamide and hyaluronic acid, which are known to limit senescence and skin aging. We conducted three different studies. (1) Ex vivo explants treated with the formula had more collagen and glycosaminoglycan. (2) In a clinical trial with forty-four women, two months of treatment improved fine lines, wrinkles, luminosity, smoothness, homogeneity, and plumpness. (3) In a third study on thirty women, we treated one arm for two months and took skin biopsies to study gene expression. 101 mRNAs and 13 miRNAs were differentially expressed. We observed a likely senomorphic effect, as there was a decrease in many SASP genes including MMP12 and CXCL9 and a significant downregulation of autocrine signaling genes: S100A8 and S100A9. These pharmaco-clinical results are the first to demonstrate the senomorphic properties of an effective anti-aging formula in skin.

## Introduction

Aging is a multifaceted process, concerning many biological phenomena, including chromosome integrity, inflammation, and cellular senescence, implicating most parts of the body^1^. Senescent cells (SCs) are characterized by irreversible cell cycle arrest and a senescence-associated secretory phenotype (SASP)^2,3^, including pro-inflammatory cytokines, MMP12 that degrades skin structure^4^, and the ‘damage associated molecular pattern’ (DAMP) secreted molecules S100A8 and S100A9^5^. Additionally, SCs disrupt the balance between cell proliferation and cell death, leading to the accumulation of damaged cells and impaired tissue regeneration. Many findings suggest that elimination of senescent cells, by senolytic compounds, or reduction of the SASP, with senomorphic molecules, have beneficial effects on aging and lifespan. Other factors, such as ultraviolet (UV) overexposure, neuropeptides, neurohormones and hormone decline, are also involved in skin aging^3,6^. Indeed, melatonin and its metabolites decrease during aging. These molecules possess antioxidant, anti-inflammatory, immunomodulatory, and anti-tumor properties. Active forms of vitamin D3 and lumisterol derivatives also decline during skin aging^7^. These vitamins are known to prevent, attenuate, or treat premature skin aging, through immunomodulation, antioxidative responses, and DNA repair mechanisms^8^.

Here we study a formula that includes several ingredients purported to act on cellular aging. The main active ingredient is 6% niacinamide, a.k.a Vitamin B3. This molecule has many beneficial effects, including reducing the harmful SASP and slowing entry of cells into senescence in vitro. Indeed, niacinamide can reduce inflammation induced by environmental stressors and it helps prevent premature aging and maintain the skin’s overall homeostasis^9^. It also attenuated expression of the aging phenotype and increased the replicative lifespan of normal human fibroblasts^10^, possibly by reducing mitochondrial activity and ROS production^11,12^. Niacinamide also increased the proliferative regenerative capacity of human primary keratinocytes; it increased clonogenicity and enriched for human stem cell ‘holoclones’^13,14^.

Another important ingredient of the formula used was hyaluronic acid (HA). HA is sorted into different molecular weight fractions and the formula contains 0.1% high molecular weight > 1 mega Dalton HA (HMW-HA) and 0.1% intermediate length fragments of around 120 kDa (HAFi). HMW-HA increases elasticity and hydration and reduces wrinkles^15^. HAFi likely has a more active action on the cells of the skin, and like niacinamide, HAFi induces keratinocyte proliferation in human skin^16^. Moreover, clinically, topical application of HAFi resulted in epidermal hyperplasia and it restored atrophic human skin to normal thickness. This effect was accompanied by significant clinical improvement, suggesting that HAFi could be therapeutic for atrophy. Indeed, more recently, topical application of intermediate size hyaluronic acid significantly improved skin quality in dermatoporosis patients and reduced the number of p16^Ink4a^-positive cells in the dermis and epidermis, suggesting an anti-senescence activity^17^. Here we perform, (1) ex vivo, (2) observational and (3) genomic cohort, studies to evaluate the senomorphic and anti-aging efficacy of an original formula containing niacinamide, HMW-HA and HAFi.

## Results

### An active formula reinforces dermal matrix ex vivo

**Figure 1:**
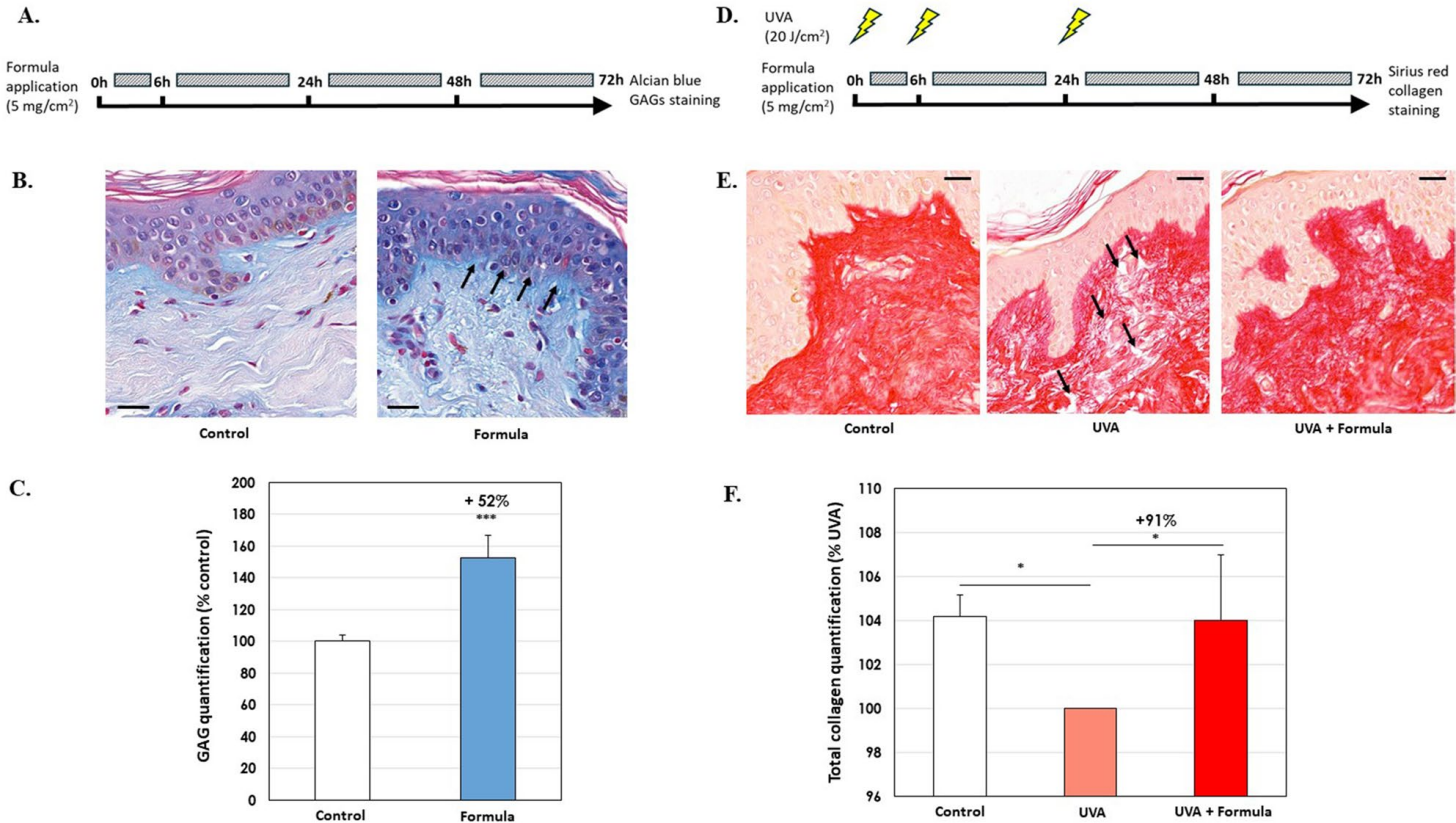
Effect of formula on dermal matrix densification. (**A**) Experimental procedure, (**B**) representative images and (**C**) quantification of the matrix glycosaminoglycan content by Alcian blue staining after 72 h of topical treatment with the formula, from three donors. *** *p* < 0.001 significantly different from control. (**D**) Experimental procedure, (**E**) representative images and (**F**) quantification of the total collagen content, by Sirius red, after chronic UVA exposure and 72 h of topical treatment with the formula of the same tissues. *p < 0.05 significantly different from the UVA-stressed control (ANOVA followed by a Dunnett post-test). Note formula shields the effect of UVA exposure. Scale bar = 20 µm.

Initially we sought to test for any physiological effects of the formula using an ex vivo model. Skin was taken from three abdominoplasty patients, who gave consent for use of their waste tissues. Explants were maintained in culture, and the formula to be tested was applied over three days (Fig. 1A). Matrix densification was evaluated histologically by glycosaminoglycan staining with Alcian blue dye (Fig. 1B). In untreated explants, glycosaminoglycans were mainly located at the dermo-epidermal junction, as a diffuse blue line just beneath the epidermis. After repeated treatment with formula, the blue color was more intense, and coloration was more uniform, starting from the epidermis and diffusing through the papillary dermis. The formula increased skin density with 52% more glycosaminoglycans (Fig. 1C, *p* < 0.001).

We therefore sought to test for an effect on skin aging by inducing photoaging in the explants. UVA exposure is known to ‘age’ skin by degrading and downregulating the extracellular matrix network, especially dermal collagen^15^. We treated the explants in parallel for three days with 20 J/cm^2^ UVA. We performed this experiment in three conditions: a control without UV, with UV only and finally with both UV and formula (Fig. 1D). Total dermal collagen was specifically stained with Sirius red dye (Fig. 1E). As expected, chronic UVA reduced collagen content and disrupted the collagen network in the papillary dermis, visualized by more diffuse staining and a much less intense red dotted pattern. Treatment with the formula, improved recovery of dermal density, and protected total collagen content. Indeed, the organization of the total collagen network, of skin that had been irradiated and then formula-treated, was almost identical to that of the non-irradiated control condition, i.e. the formula conferred complete recovery and protection from UVA (Fig. 1F). In conclusion, these ex vivo human skin experiments demonstrate anti-aging and dermal densification properties of the formula.

### Formula improves facial skin

Next, we tested the effects of the formula on facial skin quality, as per its intended use. The 44 participants were healthy woman volunteers, aged between 38 and 55 years, with a skin phototype (Fitzpatrick scale) between I and III, who gave written informed consent before application of the formula in the study. All enrolling subjects declared face skin sensitivity. All participants also presented loss of firmness and dull and uneven complexion, and a minimum level 2 on the crow’s feet wrinkle Bazin scale^18^. The women applied the formula once every morning, on the entire face, neck and neckline, for 8 weeks. Participants did not use any other facial antiaging products during the study.

**Figure 2:**
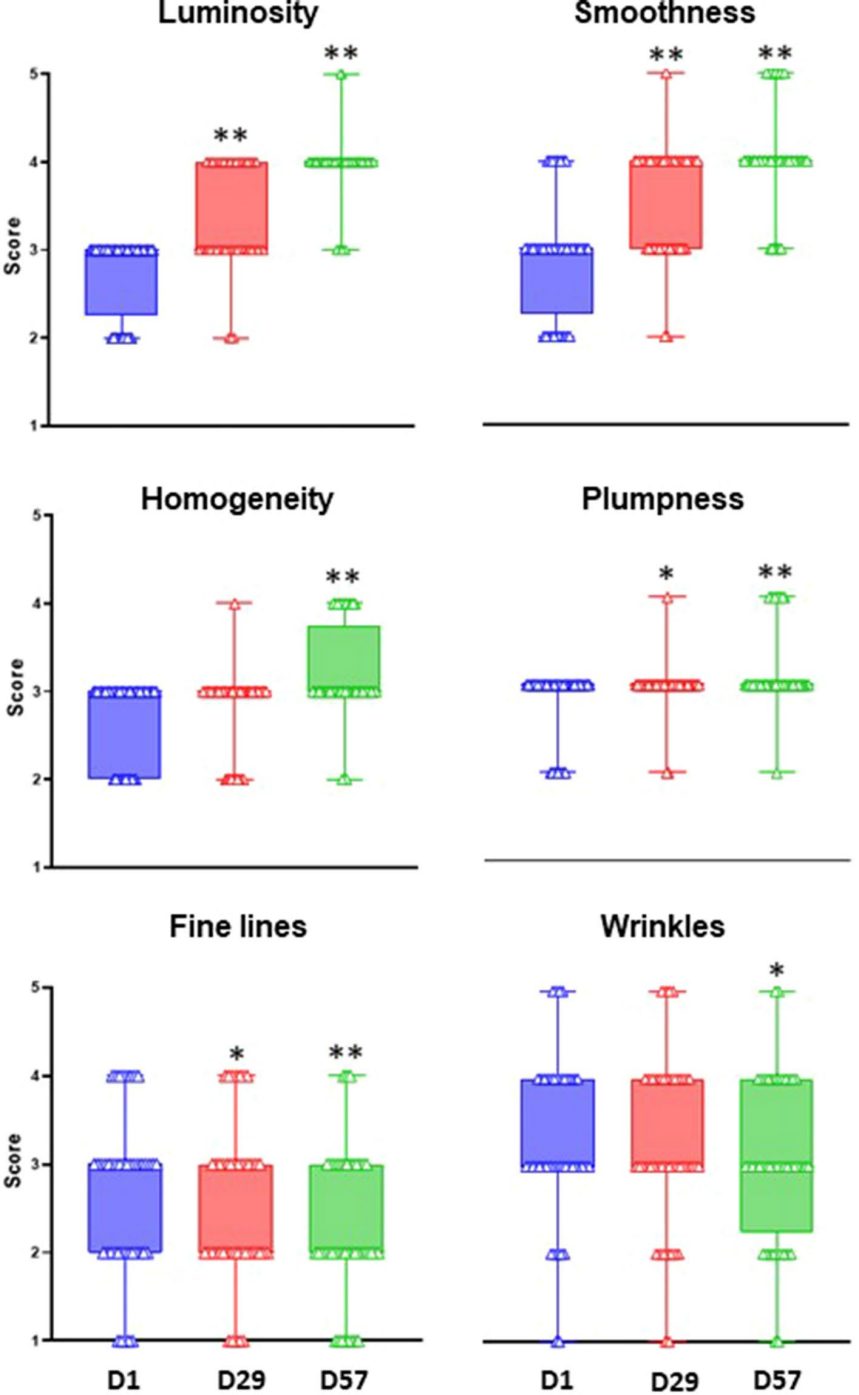
Clinical study of the effect of the formula on the face. 44 women applied the formula for two months and the six skin health parameters shown were evaluated at one-month intervals: start (D1), blue; one month (D29), red; two months (D57), green. *p < 0.05, **p < 0.01 significantly different from D1.

After daily application of the investigational product, we used six main efficacy assessments to clinically score skin aspect and texture: at baseline, after 1-month (D29) and finally at 2-months (D57). Wrinkles, fine lines, skin smoothness, skin plumpness, radiance of complexion (luminosity) and homogeneity of the skin were scored from 0 to 5 at the three time-points. There were highly significant improvements in skin quality after one and two months (Fig. 2, raw data in Supplementary Table 1). By the end of the study, all but one participant had more radiant skin (*p* < 0.0001, Wilcoxon test). Radiance increased by 44% after two months, and average radiance had increased by 21% after just one month (*p* < 0.0001). Indeed, 64% of participants had smoother skin after just one month, and all but two of the 44 participants had smoother skin after two months (*p* value < 0.0001, at both time points). Skin smoothness scores increased 39% on average. Homogeneity and plumpness increased in 43% and 39% of participants, respectively (both *p* < 0.0001). In contrast, intensity of fine lines fell on average by 15% and wrinkles were reduced in 20% of the women (*p* < 0.004).

### Formula affects aging related gene expression

**Figure 3:**
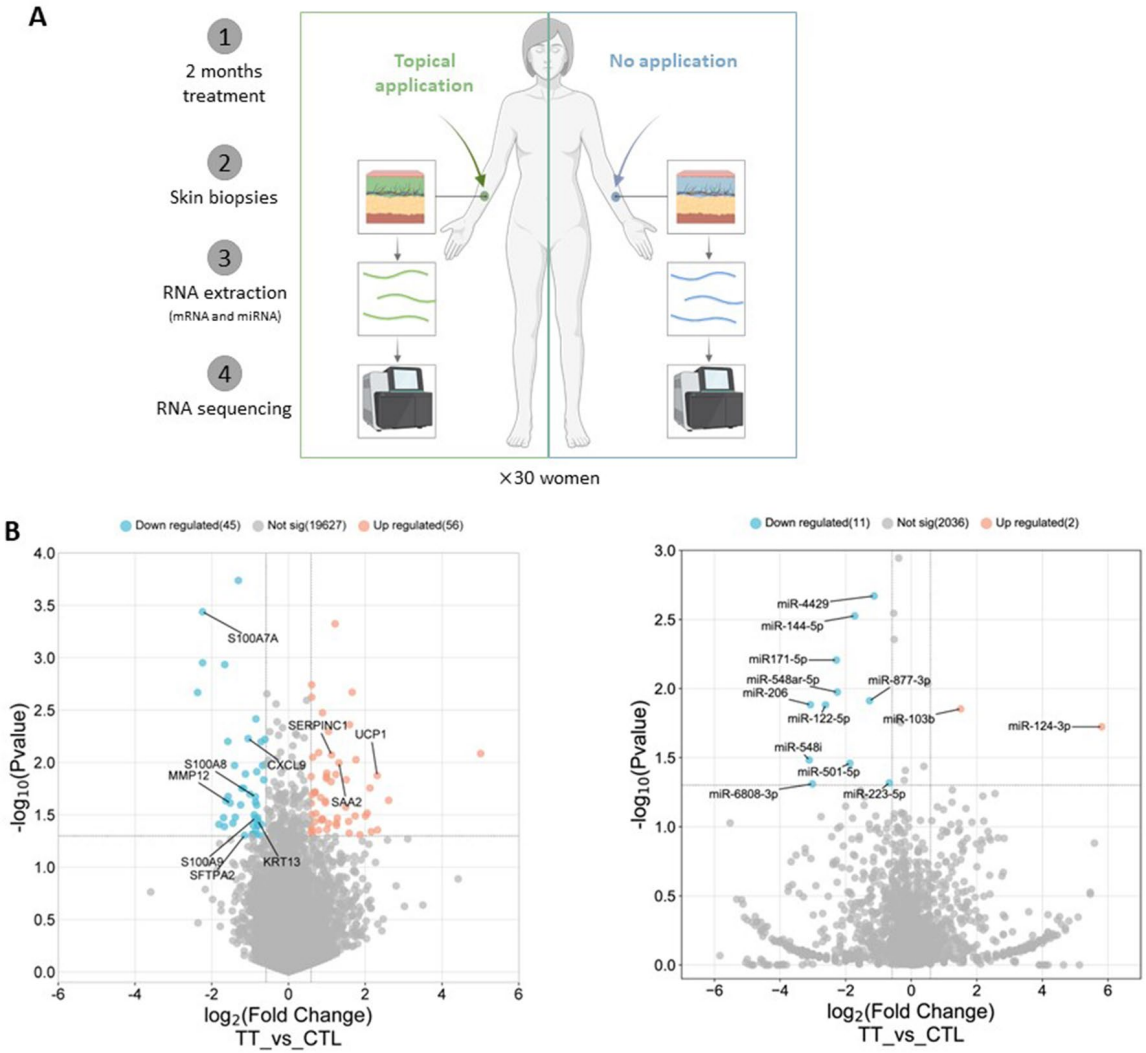
Clinical study of the formula effects on gene expression. (**A**) Study design. For thirty women, formula was applied to one arm for two months and biopsies were taken for gene expression analysis by NGS-RNAseq. (**B**) 101 mRNA genes and 13 miRNAs were differentially expressed between treated and control samples with a fold change ≥ 1.5 and with a *p* value < 0.05. Down regulated genes are shown in blue and upregulated genes are shown in orange. For tidiness, just the SASP and aging-related mRNAs are labeled.

The formula contains putative senomorphics, niacinamide and hyaluronic acid, that might improve skin quality by affecting genes involved in aging and senescence. We therefore tested if formula affects gene expression by taking small biopsies from treated and non-treated arms of thirty participants in a separate clinical study. In this third study, the formula was applied, on a designated randomized forearm, once a day for two months, according to normal conditions of use. Small biopsies were taken, RNA was extracted, and gene expression was analyzed by next-generation sequencing to compare the effect of the formula on treated and untreated arms (Fig. 3A). We separately sequenced not only mRNA, but also micro RNAs, as these two types of genetic material have various disparate roles in aging^19^. The generated data sets are provided in Supplementary Table 2. The expression levels of 101 mRNA genes and 13 miRNAs were altered more than 50% by formula, with a *p* value < 0.05. Ten of the most formula-altered genes were SASP or aging-related (Fig. 3B). A total of 45 mRNA genes were downregulated and 56 were upregulated by these strict criteria. For the differentially expressed miRNAs, 11 were downregulated and just 2 were upregulated.

### Formula-affected mRNAs and miRNAs form a potential network

**Figure 4:**
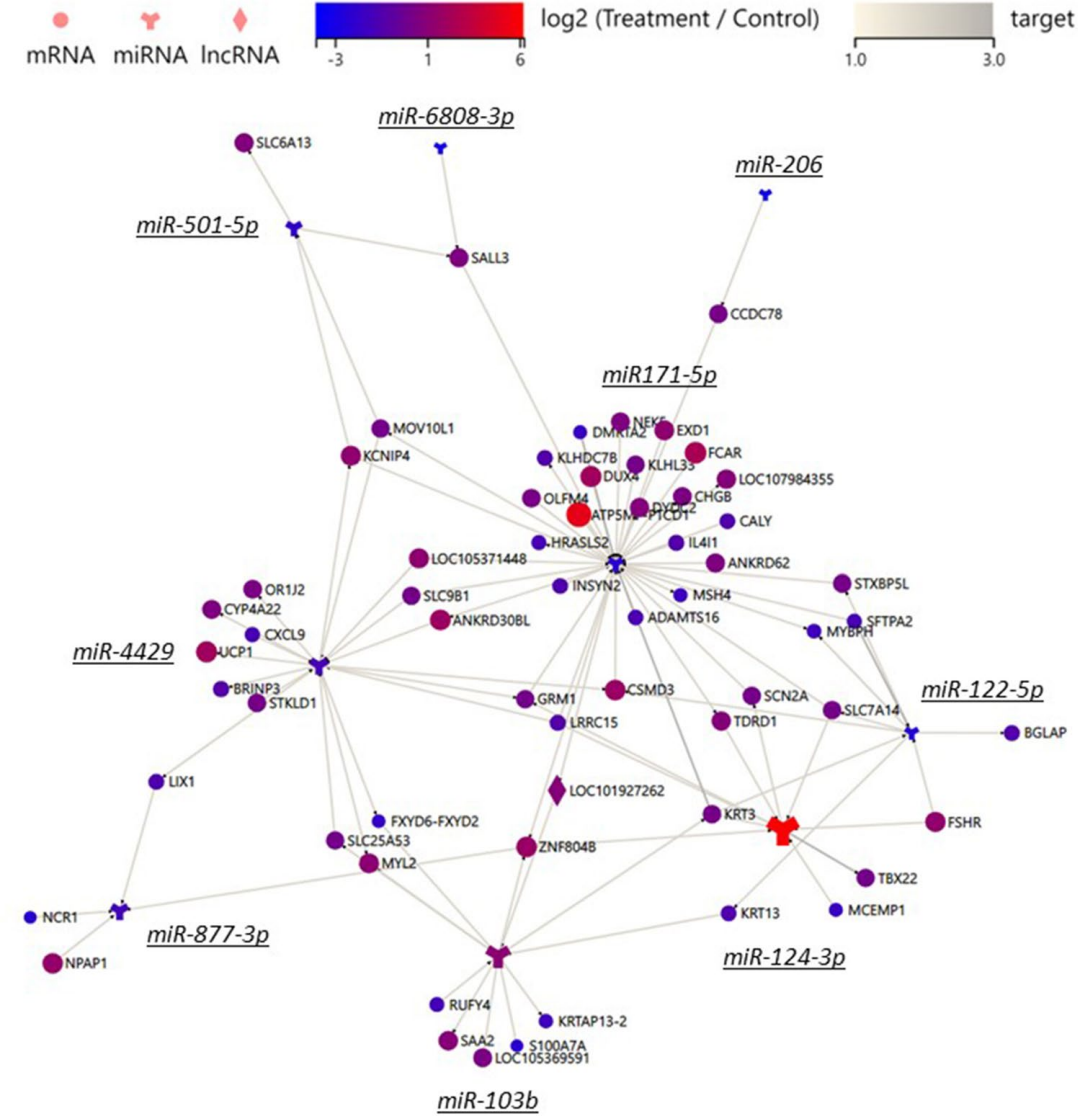
A mRNA-miRNA network is regulated by the formula. Prediction of target relationship between 13 miRNAs and 101 mRNA/lncRNAs that were significantly altered by treatment found 97 interactions between 9 miRNA and 61 mRNAs. Larger nodes indicate greater fold change.

The miRNAs that especially captured our attention were miR-206, that was reduced, and miR-124-3p, that was increased by treatment. These miRNAs have important roles in aging. Indeed, downregulation of miR-206 alleviates H202-induced senescence^20^ and miR-124-3p overexpression protects against oxidative stress and inflammation^21^. Another miRNA, miR-122-5p, that was downregulated by the formula tested here was also downregulated by acitretin treatment of psoriasis vulgaris^22^. The 13 differentially expressed miRNAs and 101 mRNAs were subjected to TargetScan, RNAhybrid and miRanda analysis to form a likely network between nine of the miRNAs and 61 mRNAs (Fig. 4; Supplementary Table 3).

To further investigate the biological processes altered by the formula we performed GO analysis on the 249 genes that were differentially expressed *p* < 0.05, while reducing the fold-change requirement to > 1.2X, as the original 1.5X requirement is likely too rigorous in a tissue containing multiple cell types. We tested the 127 downregulated and 122 upregulated genes separately and the corrected significant biological processes are shown in Table 1. Importantly, there was a 100% switch between the up- and down-regulated genes, in terms of GO process enrichment. In the downregulated genes there were only positively enriched processes, whereas in the upregulated gene set all the altered GO processes had a negative sign to the enrichment. Overall, this indicates that all the significantly altered processes in the first column of both the upper and lower parts of Table 1 were reduced, and none were increased, by the formula. The most striking process was 68-fold enrichment of autocrine signaling genes in the downregulated gene set: exemplified by S100A8, S100A9 and SERPINB3.

**Table 1:** GO ontology analysis of 127 downregulated and 122 formula-upregulated genes.

Formula-altered GO ‘biological process’	Homo sapiens	Found	Expected	Fold enrichment	Raw *p* value	FDR
Biological Processes altered in the 127 downregulated genes, p < 0.05						
autocrine signaling	7	3	0.04	+ 67.89	2.80E−05	3.63E−02
neutrophil chemotaxis	80	7	0.51	+ 13.86	1.25E−06	9.72E−03
neutrophil migration	91	7	0.57	+ 12.18	2.80E−06	8.70E−03
granulocyte migration	100	7	0.63	+ 11.09	5.04E−06	9.78E−03
myeloid leukocyte migration	138	8	0.87	+ 9.18	3.94E−06	8.75E−03
granulocyte chemotaxis	86	7	0.54	+ 12.89	1.97E−06	7.64E−03
leukocyte chemotaxis	149	8	0.94	+ 8.50	6.75E−06	1.05E−02
killing of cells of another organism	112	7	0.71	+ 9.90	1.02E−05	1.44E−02
biological process involved in interspecies interaction between organisms	1551	28	9.79	+ 2.86	4.22E−07	6.56E−03
response to other organism	1371	24	8.66	+ 2.77	5.53E−06	9.55E−03
response to external biotic stimulus	1374	25	8.67	+ 2.88	1.73E−06	8.94E−03
response to biotic stimulus	1419	25	8.96	+ 2.79	3.04E−06	7.88E−03
Biological Processes altered in the 122 upregulated genes, p < 0.05						
macromolecule metabolic process	5864	12	32.18	− 0.37	7.89E−06	4.08E−02
organic substance metabolic process	7624	19	41.84	− 0.45	3.30E−06	5.13E−02
metabolic process	8056	21	44.21	− 0.48	3.97E−06	3.09E−02

Formula-altered biological processes according to geneontology.org Panther release 18. The first column shows the biological processes that are altered in the 127 and 122, down- or up-regulated genes, at the top and bottom respectively. Columns 2–4 show the total number in the genome, number found, and number expected. Column 5 shows formula-induced fold-enrichment. Note that the fold enrichment is positive in all the downregulated processes in the top part, meaning that these processes, including autocrine signaling, are all downregulated by formula. Note also that the sign of fold-enrichment is negative throughout in all the upregulated genes, indicating that these processes are also downregulated by formula. Raw and Benjamini–Hochberg corrected *p* values are given in the final columns.

**Figure 5:**
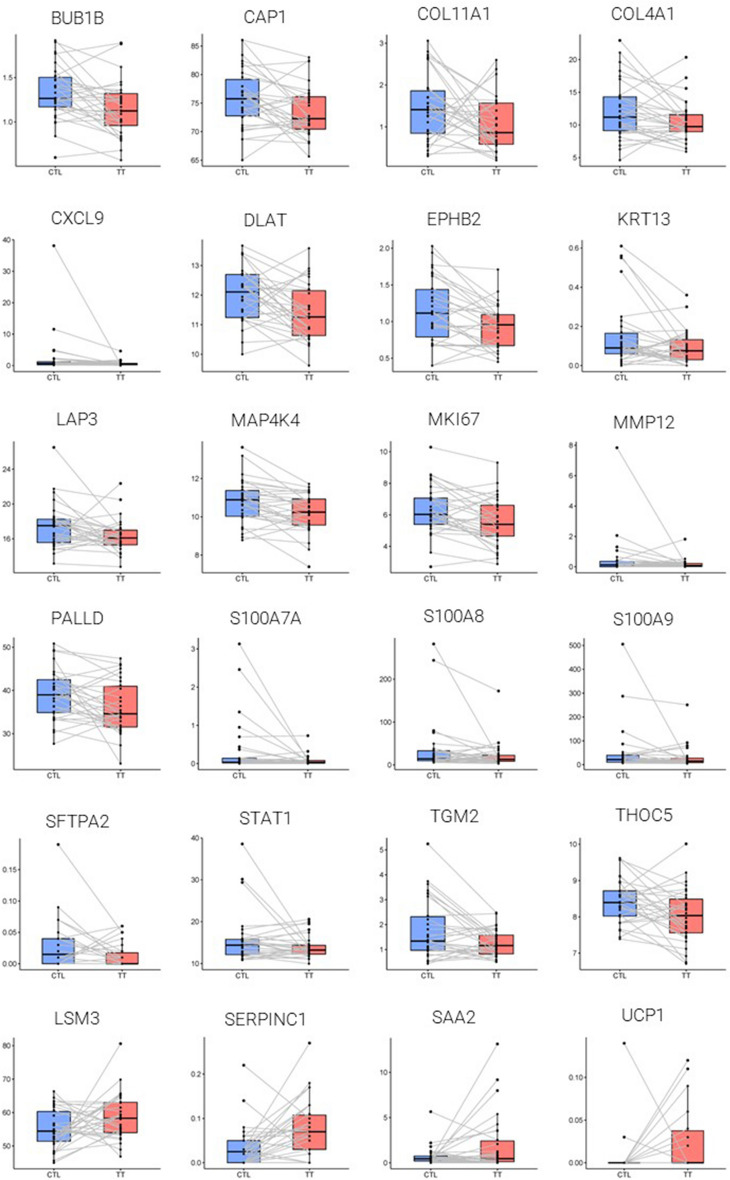
Twenty-four SASP and aging-related genes downregulated by the formula. Box plots showing expression of 24 genes belonging to the aging and SASP databases (Aging Atlas^23^, Basisty et al.^24^) with *p* value < 0.05 (Supplementary Table 2). Control and treated groups are in blue and red respectively with median and quartiles shown by the boxes. Individual patient readings, before and after treatment, are joined by lines. FPKM levels are shown on the Y axes.

To pinpoint the pathways affected by formula in more depth we extracted 2,238 genes from the aging and SASP databases^23,24^ and tested the overlap of significantly affected genes. 24 genes of these aging-related and SASP genes were altered by formula (*p* < 0.05—Supplementary Table 2). Strikingly 20 of these genes were downregulated by treatment and only four were upregulated (Fig. 5). For example, the key photoaging related gene MMP12 was downregulated by the formula. MMP12 is a matrix metalloprotease that degrades ECM and elastin^25^ and is involved in integrin signaling and dermatitis. This gene is upregulated by UVA, which causes photoaging^26^. Therefore, downregulation of MMP12 by formula could have antiaging effects. S1007A, S100A8 and S100A9^27^ were all downregulated. S100A7 is induced by telomere dysfunction^28^ and S100A8 and A9 are involved in Psoriasis vulgaris^29^. S100A8 cytokine is also a key senescence gene^30^. Like S100A8/9, TGM2 is elevated in psoriasis patients^31,32^. Similarly, also, the key aging clock chemokine CXCL9, which is involved in age-related chronic inflammation, was downregulated by formula^33^. CXCL9 is upregulated in morphea dermatitis^34^. Key Actin-binding genes CAP1 and PALLD were downregulated. The actin cytoskeleton is involved in giving senescent cells their typical flat morphology^35^. Cytokeratins form intermediate filaments in epithelial cells and the cytokeratin KRT13 was also downregulated by formula. This gene is involved in non-cornified stratified squamous epithelium^36^ and it is also involved in G1/S phase transition^37^. Indeed, various SASP/aging-related genes involved in mitosis were downregulated, e.g. BUB1B mitotic checkpoint kinase and the mitotic marker gene MKI67 (Ki-67) ^38,39^. MAP4K4 is found upregulated in Alzheimer’s disease^40^. Downregulation of all these genes is therefore consistent with a molecular antiaging effect of the formula.

Furthermore, all but two of the 20 downregulated aging and SASP genes (Fig. 5) have a positive role in aiding and abetting cancer, indicating that their downregulation by formula may assuage a potential tendency to aberrant growth in aging skin. Many of these 18 downregulated genes have specific roles in maintenance of cancer stem cells. These include cytokeratin KRT13^41,42^, CAP1^43^, PALLD^44^ TGM2^45,46^ STAT1^47^, EPHB2^48^ and S100A9^49^. BUB1B is associated with chromosomal instability and various kinds of cancer^38^. CXCL9 is associated with tumor density and MKI67 expression^50^. MKI67, also known as proliferation marker Ki-67, is the subject of hundreds of papers chronicling its involvement in numerous kinds of cancer. For example, it correlates with TP53 mutation^51^. STAT1 is a known oncogene in many cancers including endometrial cancer^52^. DLAT is a poor prognosis indicator in many types of cancer^53^. LAP3 promotes invasion of breast cancer cells by upregulating metalloproteases^54^. MMP12 is a poor prognostic indicator for six different types of cancer^55^ including melanoma^56^. MAP4K4 promotes ovarian cancer metastasis^57^. Downregulation of many of these genes by formula could thus have pleiotropic effects combining a positive effect in delaying the cancer stem cell properties that increase with aging.

We were surprised to see two collagens in the list of downregulated genes as we were expecting formula to increase overall collagen, as we had observed in Fig. 1. However these two minor downregulated collagens are indeed both known to be involved in cancer: COLA4A is upregulated by p53^58^ and COL11A1 is a biomarker of invasive carcinoma-associated cells^59,60^. Indeed, close inspection of our data revealed that these two collagens likely have specialised functions, as overall we detected 46 COL genes in our RNA seq data (Supplementary Table 2) of which the most abundant were COL1A1, COL6A2, COL17A1, COL1A2, COL6A1, COL3A1 in that order that together made up 90% of the total collagen FPKM counts. However, the two downregulated collagens, COL4A1 and COL11A1, made up less than 0.06% of the total collagen content between them, so their increase in no way reflects a reduction of total collagen.

There was clearly some heterogeneity in the expression, of the genes that formula downregulated, and for several of these SASP genes, there was one participant who had extremely high expression in the untreated condition, that was completely abrogated in the treated condition. For example, participant Number 10, had the highest levels for the three S100A genes (7A, 8 and 9), whereas the level of these genes was reduced to near zero in all cases by the formula. Similarly, for both CXCL9 and MMP12, a different participant, Number 2, had high levels that were reduced to close to zero by formula. Overall, these data, demonstrating that twenty aging and SASP-related genes were downregulated, are consistent with a beneficial effect of the formula on the very causes of physiological aging, more specifically with a clearing effect, where aberrant high expression of SASP genes is screened by the formula to maintain healthy skin.

## Discussion

This is the first study to describe a clinically effective senomorphic effect of a topically administered product. Our aim was to test the molecular effects of a unique cosmetic formulation, containing two active ingredients, with previously demonstrated in vitro senomorphic activity: 6% niacinamide and 0.1% medium weight hyaluronic acid fragments (HAFi). This is also the first study using transcriptomics to demonstrate a clinical senomorphic effect of a topically administered product. These molecular effects were correlated with reduction of wrinkles and fine lines and an enhancement of skin radiance.

In the first of three different studies we used ex vivo explants; in the second we tested the formula on the face and in the third study, we compared gene expression, between treated and control arms. All the studies gave significant results. In the ex vivo study, the formula upregulated collagen and glycosaminoglycan (Fig. 1). When applied to face and neck, the formula improved skin texture and aspect by decreasing wrinkles, fine lines, and improving skin smoothness, plumpness, radiance complexion and skin homogeneity (Fig. 2). The improvements were clearly progressive, and results were visible after one month and better after two months. Quantitatively, radiance for example, was 21% higher after one-month and 42% better at two-months, so it could be reasonably assumed that treating for three or four months would lead to further improvement. Improvements were also observed for face smoothness: up 22% after one month, and 39% higher at two months. Similarly, in the study of gene expression after two months, we started to notice differences, in terms of the decrease of aging- and SASP-associated genes (Table 1, Figs. 3 and 5).

Indeed, Table 1 demonstrates a concerted downregulation of SASP-associated functions, like autocrine signaling, by formula. In Fig. 5 we identify many downregulated genes that may collaborate as part of a network. For example, STAT1 and TGM2 that inhibits the degradation of STAT1^61^ that upregulates MKI67^62^, so they are likely all downregulated in concert by the formula. We also identified several potential interactions between miRNA targets and mRNAs. For example, hsa-miR-103 is upregulated by treatment and correlates negatively with STAT1, which is downregulated by formula^63^.

In terms of the mechanism for the robust improvements in skin quality observed here, there are some effects on SASP and aging-related gene expression that remain to be elucidated. Clearly the use of macroscopic skin biopsy is a global strategy that incorporates all types of cells, including blood cells and keratinocytes in the epidermis, and a much smaller proportion from the dermal layer where fibroblasts, stem cells and senescent cells reside^35^. These different cell types have non-exclusive overlapping expression of most aging- and SASP-related genes so that differences in expression of key stem cell genes, for example, may be masked by other cell types. One way around this problem would be fluorescence-activated cell-sorting, with known markers of the different cell types, or employing single cell RNAseq. Another limitation is the brevity of the study, where it would be good to study a larger cohort over a longer period. We suspect that after just two months of treatment we are just seeing the beginning of the beneficial long-term changes in gene expression caused by the formula. Finally, while we studied mRNA and miRNA changes, epigenetic changes, i.e. DNA methylation and histone acetylation, would also warrant scrutiny.

Antiaging ingredients and treatments are currently the subject of intense research^3^. Another putative anti-aging formula containing *Harungana madagascariensis* extract found MKI67 was upregulated in cell culture^64^. This difference in this ubiquitous cell division marker could be due to the active ingredients or the fact that ours was a pharmaco-clinical study on human subjects and not on cell lines. The latter hypothesis is corroborated by the fact that the senescence marker p16INK4A is upregulated in both dermis and epidermis in the elderly, but that p16INK4A and MKI67 were expressed mutually exclusively, in different cell types^65^. Indeed, the fact that both keratinocytes and fibroblasts can become senescent, means it is possible that one formula works more on one cell type, while having the opposite effect on the other^66^. Each cell type has a perfect complex balance between healthy cell division, excessive cell division or even cancer and the arrested cell division of senescent cells^35^. In conclusion then, this is the first pharmaco-clinical study to use genome wide analysis to demonstrate senomorphic properties of an anti-aging formula.

## Materials and methods

The current study tested a dermocosmetic formulation. According to International nomenclature of cosmetic ingredients, the full ingredients were: Avene thermal spring water, caprylic/capric triglyceride, glycerine, niacinamide, *Carthamus tinctorius* seed oil, *Butyrospermum parkii* butter, glycol palmitate, arachidyl alcohol, cetearyl alcohol, cellulose, glyceryl stearate, sodium hyaluronate, adenosine, arachidyl glucoside, behenyl alcohol, caprylyl glycol, cetearyl glucoside, citric acid, *Terminalia chebula* fruit extract, fragrance, *Helianthus annuus* seed oil, sodium benzoate, tocopherol, tocopheryl glucoside and xanthan gum. For simplicity, this “dermocosmetic formulation” is referred to as the ‘formula’. The formula was tested in three different studies headlined below: ex vivo histology, intended use physiology and biopsy genomics.

### Ex vivo histology of the effect of formula on dermal matrix densification

1.12 cm^2^ skin explants were isolated from three different abdominoplasty surgery patients of 24, 25 and 36 years of age. The donors provided written donor consent beforehand according to the French legal requirements on donor rights. Explants were excised and seeded in polycarbonate inserts of a 6-well plate containing a survival medium, based on DMEM, supplemented with antibiotics and an antifungal agent. This ex vivo organ culture system maintained the skin at the air–liquid interface allowing dermis and epidermis to be supplied through nutrient diffusion across the insert. Explants were then incubated at 37 °C in a humid atmosphere with 5% CO_2_. The formula was applied at 5 mg/cm^2^, at times 0, 6, 24 and 48 h, as per a previously published protocol^67^. Skin was harvested, 24 h after the four treatments, for glycosaminoglycan semi-quantification, using Alcian blue histological staining. To induce photoaging stress to the skin, we followed the same regimen as above, but included three 365 nm UVA doses of 20 J/cm^2^, using a Biosun Vilber Lourmat simulator (Vilber, Eberhardzell, Germany), immediately before the first three formula applications, as previously described^68^. Skin was then harvested as above and quantified for total collagen by Sirius red histological staining.

### Physiology of formula activity in its intended use on the face

We present a noncomparative, open label monocentric study, according to the following definitions. ‘Non comparative’ indicates a study design where each subject is treated with an active formula and followed over the time, without untreated control humans. ‘Open label’ indicates that the subjects know that they are receiving an active non-placebo formula, without knowing anything about its ingredients or provenance. ‘Monocentric’ indicates a clinical study performed in a single clinic. This 2-month clinical study was carried out at the facilities of Insight Research, Quatre Bornes, Mauritius, according to the ethical principles stated in the Declaration of Helsinki and its subsequent amendments (1964, and last amendment in force), and in accordance with the guidelines for Good Clinical Practices (CPMP/ICH/135/95) published by the International Conference on Harmonization (adopted by CHMP, 15/12/2016, issued as EMA/CHMP/ICH/135/1995). There were 44 women in the study with a mean age of 48 years (min 38, max 55). Of the 44 women: 10 women had type II phototype and 34 women had III phototype on the Fitzpatrick scale. The women presented a mixture of skin types: 6 (14%) were normal, 5 (11%) were dry, 9 had greasy skin (20%) and 24 (55%) had a combination of the above. The women applied formula to their faces daily for two months. At one-month intervals, they were assessed by a dermatologist from Insight Research, Mauritius, on a scale of zero to five, for skin health parameters: luminosity, smoothness, homogeneity, plumpness, fine lines and wrinkles. The one-month time-point was flexible between day 29–31 and the two-month between day 57–60, with the condition that the product was used daily throughout the trial period up until the day before each assessment. Data were analyzed by paired t-test.

### Effect of the formula on gene expression in biopsies

Thirty women, aged from 35 to 55 years, took part in this trial of daily application of the formula on one of their arms over two months (ClinicalTrials.gov identifier, NCT number: NCT05895591, first posted date 08/06/2023). This study was conducted by Spincontrol (Tours, France) between September and December 2021, in accordance with the Declaration of Helsinki (1964) and with Good Clinical Practice guidelines. In agreement with the French law (ordonnance no. 2016-800, 16/06/2016 and decree no. 2017-884, 09/05/2017), this study was not required to be submitted to an ethics committee as it evaluates a cosmetic product. All volunteers provided written and informed consent. All samples were anonymized before analysis.

For biopsy harvesting, local anesthesia was carried out on the external side of, both the treated and untreated, forearms using injectable anesthetic Xylocaïne® (20 mg/ml). A waiting period of 3–5 min is required for the anesthetic to take effect. A total of four samples were taken: two from each side, i.e. two treated and two control samples, with a 3.5 mm disposable biopsy punch. After sampling, a Steri-Strip™ was put on biopsy areas to ease the healing. Cutaneous biopsies were immediately stored at − 20 °C in RNAlater (Qiagen).

Total RNA was extracted using miRNeasy Tissue/Cells Advanced Mini Kit (# 217604, Qiagen) according to the manufacturer’s instructions. RNA quality was assessed on a Nanodrop (ThermoFisher) and High Sensitivity RNA Screen Tape (Agilent 2200 TapeStation system) was used to determine RNA integrity and the rRNA ratio. mRNA and miRNA sequencing were performed on the DNBSEQ sequencing platform by BGI Genomics (Hong Kong). Briefly, after RNA enrichment and purification, libraries were constructed by adding unique adapters for sample multiplexing at 10 samples per flow cell. Samples were then sequenced: paired-end 150 bp for mRNA/lncRNA, and single-end 50 bp for miRNA. After quality control, the filtered clean reads were aligned to Human reference sequence genome version: GCF_000001405.38_GRCh38.p12, with HISAT and then mapped to Homo sapiens reference genes using the BOWTIE2 pipeline. Alignment quality was further checked with statistics of the mapping rate and read distribution on the reference genome before gene quantification and further downstream analysis. The DEseq2 method was used in group comparison and final gene lists^69^. Data analysis and illustration were made with the help of the following software or indicated R packages by standard procedures: Dr.Tom from Beijing Genomics Institute, bioinformatics.com.cn/en, Gene Ontology knowledgebase, release 2023-01-01/ version number 10.5281/zenodo.7504797 and BioRender.

### Supplementary Information

Supplementary Information 1.

Supplementary Information 2.

Supplementary Information 3.

## Data Availability

The data that support the findings of this study are not openly available due to reasons of sensitivity and are available from the corresponding authors upon reasonable request.
