# Supplementary material for: Senomorphic activity of a combination of niacinamide and hyaluronic acid: correlation with clinical improvement of skin aging
Source: Sci Rep. 2024 Jul 15;14:16321. doi: 10.1038/s41598-024-66624-7 (PMC11251187; doi:10.1038/s41598-024-66624-7)
Supplement: Supplementary file 1 — Supplementary Information 1. [file 41598_2024_66624_MOESM1_ESM.pdf]

Supplementary Table 1 : Clinical scoring of skin aspect and texture

	Wrinkles					Fine lines				
Subject #	D1	D29	D57	ΔD29	ΔD57	D1	D29	D57	ΔD29	ΔD57
1	3	3	3	0	0	2	2	2	0	0
2	2	2	2	0	0	1	1	1	0	0
3	1	1	1	0	0	1	1	1	0	0
4	3	3	3	0	0	2	2	2	0	0
5	3	3	2	0	-1	2	2	2	0	0
6	3	3	3	0	0	3	3	2	0	-1
7	3	3	2	0	-1	2	2	2	0	0
8	2	2	2	0	0	2	2	2	0	0
9	3	3	3	0	0	1	1	1	0	0
10	3	3	3	0	0	2	2	2	0	0
11	4	4	3	0	-1	2	2	2	0	0
12	2	2	2	0	0	1	1	1	0	0
13	4	4	3	0	-1	2	2	2	0	0
14	4	4	4	0	0	3	3	3	0	0
15	3	3	3	0	0	3	2	2	-1	-1
16	2	2	2	0	0	1	1	1	0	0
17	1	1	1	0	0	2	2	1	0	-1
18	4	4	3	0	-1	3	3	3	0	0
19	4	4	4	0	0	3	3	3	0	0
20	4	4	4	0	0	3	3	3	0	0
21	4	4	4	0	0	3	3	3	0	0
22	5	5	5	0	0	4	4	4	0	0
23	3	3	3	0	0	2	2	2	0	0
24	2	2	2	0	0	2	2	2	0	0
25	3	3	3	0	0	2	2	2	0	0
26	3	2	3	-1	0	3	3	3	0	0
27	3	3	3	0	0	3	3	2	0	-1
28	3	3	3	0	0	3	2	2	-1	-1
29	3	3	3	0	0	3	3	2	0	-1
30	3	3	3	0	0	3	2	2	-1	-1
31	3	3	3	0	0	3	2	2	-1	-1
32	4	4	4	0	0	4	4	3	0	-1
33	4	4	4	0	0	4	4	3	0	-1
34	5	5	4	0	-1	4	4	3	0	-1
35	4	4	4	0	0	3	3	3	0	0
36	4	4	4	0	0	4	3	3	-1	-1
37	4	4	4	0	0	3	3	3	0	0
38	3	2	2	-1	-1	2	2	1	0	-1
39	5	5	5	0	0	4	4	4	0	0
40	3	3	3	0	0	3	3	2	0	-1
41	4	4	4	0	0	4	4	3	0	-1
42	3	3	3	0	0	3	3	2	0	-1
43	3	3	2	0	-1	3	2	1	-1	-2
44	5	5	4	0	-1	4	4	4	0	0
**Mean**	**3.27**	**3.23**	**3.07**	**-0.05**	**-0.20**	**2.66**	**2.52**	**2.25**	**-0.14**	**-0.41**
Median	3.00	3.00	3.00	0.00	0.00	3.00	2.00	2.00	0.00	0.00
Minimum	*1.00*	*1.00*	*1.00*	*-1.00*	*-1.00*	*1.00*	*1.00*	*1.00*	*-1.00*	*-2.00*
Maximum	*5.00*	*5.00*	*5.00*	*0.00*	*0.00*	*4.00*	*4.00*	*4.00*	*0.00*	*0.00*
**SEM**	**0.14**	**0.15**	**0.14**	**0.03**	**0.06**	**0.14**	**0.14**	**0.13**	**0.05**	**0.08**
CI 95%	0.29	0.30	0.28	0.06	0.12	0.28	0.27	0.26	0.11	0.16
			P	0.5000	0.0039			P	0.0313	<.0001
			Type of statistical test	Wilcoxon signed rank test	Wilcoxon signed rank test			Type of statistical test	Wilcoxon signed rank test	Wilcoxon signed rank test
			Δ%	-1	-6			Δ%	-5	-15
			% of subjects presenting worsening	0	0			% of subjects presenting worsening	0	0
			% of subjects without change	95	80			% of subjects without change	86	61
			% of subjects presenting improvement	5	20			% of subjects presenting improvement	14	39

	Skin smoothness					Skin plumpness				
Subject #	D1	D29	D57	ΔD29	ΔD57	D1	D29	D57	ΔD29	ΔD57
1	3	3	4	0	1	3	3	4	0	1
2	4	4	4	0	0	3	4	4	1	1
3	3	3	4	0	1	3	3	4	0	1
4	3	4	4	1	1	3	3	3	0	0
5	3	3	4	0	1	3	3	3	0	0
6	3	3	4	0	1	3	3	3	0	0
7	4	4	4	0	0	3	3	3	0	0
8	2	3	4	1	2	3	3	4	0	1
9	4	4	5	0	1	3	3	4	0	1
10	3	4	4	1	1	3	3	4	0	1
11	4	4	5	0	1	2	3	3	1	1
12	2	3	3	1	1	3	3	3	0	0
13	4	4	5	0	1	3	3	3	0	0
14	3	4	5	1	2	3	3	3	0	0
15	3	3	4	0	1	3	3	3	0	0
16	2	3	4	1	2	3	3	3	0	0
17	3	4	4	1	1	3	3	3	0	0
18	3	4	4	1	1	3	3	3	0	0
19	3	4	4	1	1	2	3	3	1	1
20	3	4	4	1	1	3	3	3	0	0
21	3	4	4	1	1	3	3	3	0	0
22	4	4	5	0	1	2	3	3	1	1
23	3	4	4	1	1	3	3	3	0	0
24	2	3	3	1	1	3	3	3	0	0
25	2	2	3	0	1	3	3	3	0	0
26	3	4	4	1	1	3	3	3	0	0
27	3	4	5	1	2	3	3	4	0	1
28	3	4	4	1	1	3	3	3	0	0
29	3	4	5	1	2	3	3	4	0	1
30	2	3	4	1	2	3	3	3	0	0
31	3	4	4	1	1	2	3	3	1	1
32	2	3	3	1	1	2	3	3	1	1
33	3	4	4	1	1	3	3	3	0	0
34	4	4	5	0	1	2	2	3	0	1
35	2	3	4	1	2	3	3	3	0	0
36	2	3	3	1	1	3	3	3	0	0
37	4	5	5	1	1	3	3	3	0	0
38	3	4	4	1	1	3	3	4	0	1
39	3	4	4	1	1	2	3	3	1	1
40	2	3	4	1	2	3	3	3	0	0
41	3	3	4	0	1	3	3	3	0	0
42	2	2	3	0	1	2	3	3	1	1
43	3	4	4	1	1	3	3	3	0	0
44	3	3	4	0	1	2	2	2	0	0
**Mean**	**2.93**	**3.57**	**4.07**	**0.64**	**1.14**	**2.80**	**2.98**	**3.18**	**0.18**	**0.39**
Median	3.00	4.00	4.00	1.00	1.00	3.00	3.00	3.00	0.00	0.00
Minimum	*2.00*	*2.00*	*3.00*	*0.00*	*0.00*	*2.00*	*2.00*	*2.00*	*0.00*	*0.00*
Maximum	*4.00*	*5.00*	*5.00*	*1.00*	*2.00*	*3.00*	*4.00*	*4.00*	*1.00*	*1.00*
**SEM**	**0.10**	**0.09**	**0.09**	**0.07**	**0.07**	**0.06**	**0.04**	**0.07**	**0.06**	**0.07**
CI 95%	0.20	0.19	0.18	0.15	0.14	0.12	0.08	0.14	0.12	0.15
			p	<.0001	<.0001			p	0.0078	<.0001
			Type of statistical test	Wilcoxon signed rank test	Wilcoxon signed rank test			Type of statistical test	Wilcoxon signed rank test	Wilcoxon signed rank test
			Δ%	22	39			Δ%	7	14
			% of subjects presenting worsening	0	0			% of subjects presenting worsening	0	0
			% of subjects without change	36	5			% of subjects without change	82	61
			% of subjects presenting improvement	64	95			% of subjects presenting improvement	18	39

	Radiance of complexion (Luminosity)					Homogeneity of skin				
Subject #	D1	D29	D57	ΔD29	ΔD57	D1	D29	D57	ΔD29	ΔD57
1	2	2	3	0	1	2	2	3	0	1
2	3	4	4	1	1	2	2	3	0	1
3	3	3	4	0	1	3	3	4	0	1
4	3	3	4	0	1	2	2	3	0	1
5	3	4	4	1	1	3	3	4	0	1
6	3	3	4	0	1	2	2	3	0	1
7	3	3	4	0	1	3	3	4	0	1
8	3	3	4	0	1	3	3	3	0	0
9	3	4	4	1	1	3	3	3	0	0
10	3	4	5	1	2	3	3	4	0	1
11	3	3	4	0	1	2	2	2	0	0
12	3	3	3	0	0	3	3	3	0	0
13	3	4	4	1	1	3	3	3	0	0
14	3	4	4	1	1	3	3	3	0	0
15	3	4	4	1	1	3	3	4	0	1
16	3	4	4	1	1	3	3	4	0	1
17	3	4	4	1	1	3	3	3	0	0
18	3	4	4	1	1	3	3	4	0	1
19	3	4	4	1	1	3	3	4	0	1
20	3	3	4	0	1	3	3	3	0	0
21	3	4	4	1	1	3	3	3	0	0
22	3	4	4	1	1	3	3	3	0	0
23	3	4	4	1	1	3	3	3	0	0
24	2	3	3	1	1	2	2	2	0	0
25	3	3	4	0	1	3	3	3	0	0
26	2	3	3	1	1	3	3	3	0	0
27	2	3	4	1	2	2	3	3	1	1
28	2	3	4	1	2	2	3	4	1	2
29	2	3	4	1	2	2	2	2	0	0
30	3	3	4	0	1	3	3	3	0	0
31	2	3	4	1	2	3	3	3	0	0
32	2	2	4	0	2	2	2	3	0	1
33	3	3	4	0	1	3	3	4	0	1
34	3	4	5	1	2	3	3	3	0	0
35	3	3	4	0	1	3	3	3	0	0
36	3	3	4	0	1	3	3	3	0	0
37	3	4	4	1	1	3	3	3	0	0
38	3	3	4	0	1	3	3	3	0	0
39	3	4	4	1	1	3	3	3	0	0
40	3	3	4	0	1	3	3	3	0	0
41	3	4	4	1	1	3	4	4	1	1
42	2	3	3	1	1	2	2	3	0	1
43	2	3	4	1	2	2	2	3	0	1
44	2	2	3	0	1	3	3	3	0	0
**Mean**	**2.75**	**3.34**	**3.91**	**0.6**	**1.2**	**2.73**	**2.80**	**3.18**	**0.07**	**0.45**
Median	3.00	3.00	4.00	1.00	1.00	3.00	3.00	3.00	0.00	0.00
Minimum	*2.00*	*2.00*	*3.00*	*0.00*	*0.00*	*2.00*	*2.00*	*2.00*	*0.00*	*0.00*
Maximum	*3.00*	*4.00*	*5.00*	*1.00*	*2.00*	*3.00*	*4.00*	*4.00*	*1.00*	*2.00*
**SEM**	**0.07**	**0.09**	**0.06**	**0.1**	**0.1**	**0.07**	**0.07**	**0.08**	**0.04**	**0.08**
CI 95%	0.13	0.18	0.13	0.15	0.13	0.14	0.14	0.16	0.08	0.17
			p	<.0001	<.0001			p	0.2500	<.0001
			Type of statistical test	Wilcoxon signed rank test	Wilcoxon signed rank test			Type of statistical test	Wilcoxon signed rank test	Wilcoxon signed rank test
			Δ%	21	42			Δ%	3	17
			% of subjects presenting worsening	0	0			% of subjects presenting worsening	0	0
			% of subjects without change	41	2			% of subjects without change	93	57
			% of subjects presenting improvement	59	98			% of subjects presenting improvement	7	43
